# Supplementary figures and images for: NGS-Based Molecular Karyotyping of Multiple Myeloma: Results from the GEM12 Clinical Trial
Source: Cancers (Basel). 2022 Oct 21;14(20):5169. doi: 10.3390/cancers14205169 (PMC9601262; doi:10.3390/cancers14205169)

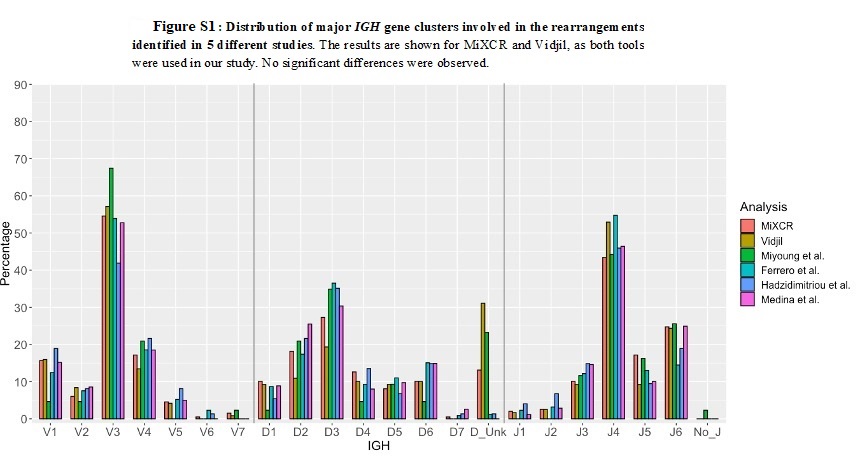

Supplement: Supplementary file 1 [file cancers-14-05169-s001.zip › cancers-1947661-supplementary/Supplementary Figure S1.jpg]

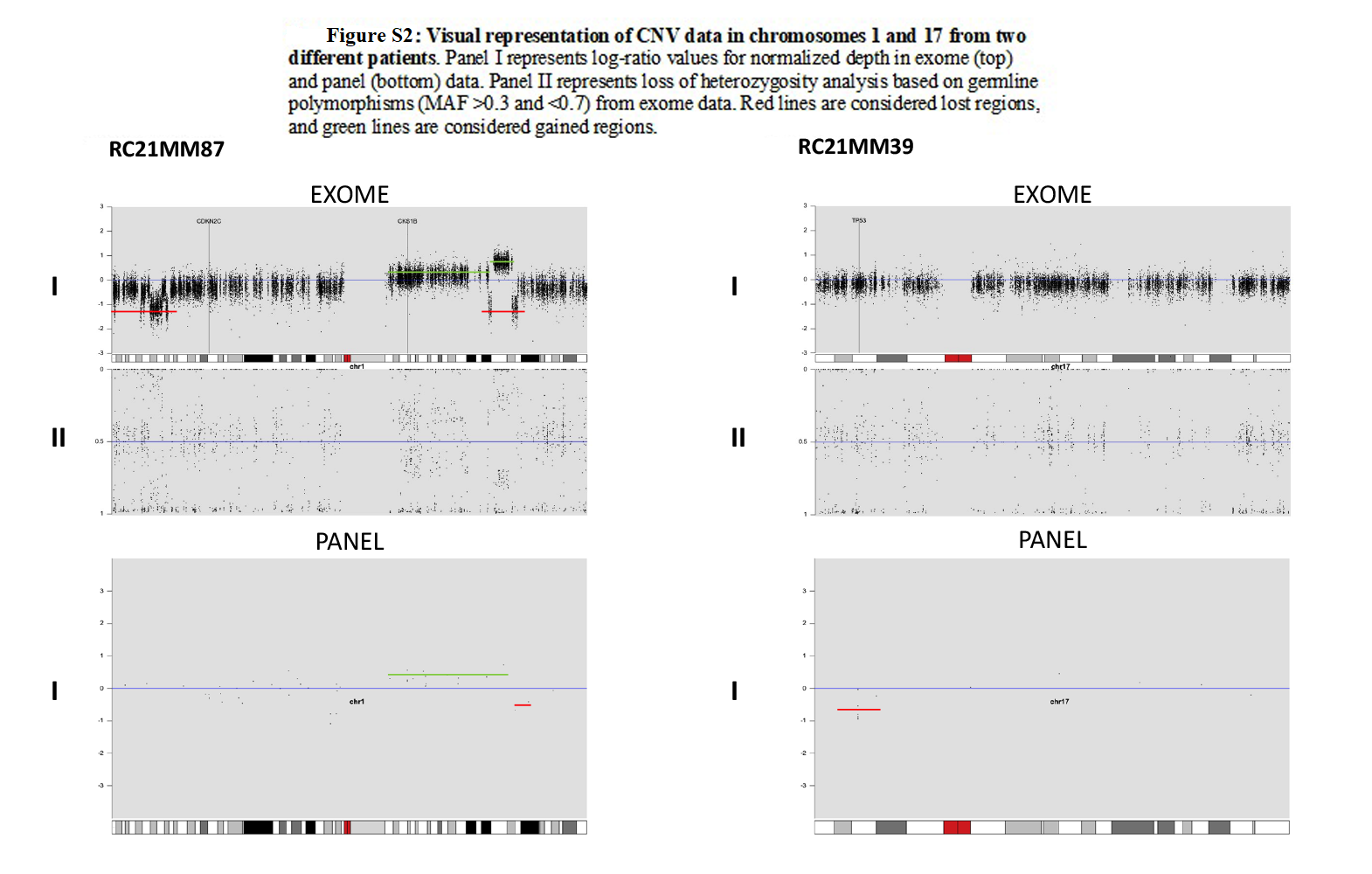

Supplement: Supplementary file 1 [file cancers-14-05169-s001.zip › cancers-1947661-supplementary/Supplementary Figure S2.tif]
